# The roles of ubiquitination and deubiquitination of NLRP3 inflammasome in inflammation-related diseases: A review

**DOI:** 10.17305/bb.2023.9997

**Published:** 2024-08-01

**Authors:** Shaokai Tang, Yuanwen Geng, Yawei Wang, Qinqin Lin, Yirong Yu, Hao Li

**Affiliations:** 1 School of Physical Education, Yanshan University, Qinhuangdao, China; 2 School of Public Administration, Yanshan University, Qinhuangdao, China

**Keywords:** Ubiquitination, deubiquitination, NOD-like receptor pyrin domain-containing protein 3 (NLRP3) inflammasomes, inflammation-related diseases, post-translational modification (PTM)

## Abstract

The inflammatory response is a natural immune response that prevents microbial invasion and repairs damaged tissues. However, excessive inflammatory responses can lead to various inflammation-related diseases, posing a significant threat to human health. The NOD-like receptor pyrin domain-containing protein 3 (NLRP3) inflammasome is a vital mediator in the activation of the inflammatory cascade. Targeting the hyperactivation of the NLRP3 inflammasome may offer potential strategies for the prevention or treatment of inflammation-related diseases. It has been established that the ubiquitination and deubiquitination modifications of the NLRP3 inflammasome can provide protective effects in inflammation-related diseases. These modifications modulate several pathological processes, including excessive inflammatory responses, pyroptosis, abnormal autophagy, proliferation disorders, and oxidative stress damage. Therefore, this review discusses the regulation of NLRP3 inflammasome activation by ubiquitination and deubiquitination modifications, explores the role of these modifications in inflammation-related diseases, and examines the potential underlying mechanisms.

## Introduction

Inflammation is a defense mechanism of the immune system against various stimuli, including microbial infections, tissue injuries, and other harmful stimuli. This process, characterized by the infiltration of numerous inflammatory biomolecules into infected and damaged areas, promotes wound healing and enhances resistance to pathogens. Persistent or excessive inflammatory responses can lead to inflammation-related diseases, such as diabetic complications, neurodegenerative diseases (NDDs), musculoskeletal system disorders, inflammatory bowel disease (IBD), and other inflammatory diseases [1]. The prevalence of these diseases has been increasing annually in recent times. Due to the complexity of their pathogenesis and causative factors, these diseases significantly affect the health and quality of life of patients [2]. The use of drugs, such as non-steroidal anti-inflammatory drugs, steroid hormones, immunosuppressants, and biological agents, is the primary clinical approach to inhibit the inflammatory response process [3]. However, prolonged use of these medications can cause diverse side effects and various comorbidities. Additionally, these drugs cannot adequately address the irreversible tissue damage, organ failure, and lifelong weakness induced by inflammation-related diseases [1]. Therefore, studying the pathological mechanisms and identifying potential therapeutic targets for inflammation-related diseases has important theoretical significance and offers valuable clinical guidance.

Inflammasomes are essential multiprotein complexes that play a critical role in host defense against pathogen invasion, physiological aberrations, and the recognition of self-danger signals. Inflammasome complexes are composed of pattern-recognition receptors (PRRs), apoptosis-associated speck-like proteins (ASC) domain containing a caspase activation and recruitment domain (CARD), and cysteinyl aspartate specific proteinase-1 (Caspase-1) [4]. During a natural immune response, inflammasomes are assembled by PRRs that recognize danger-associated molecular patterns (DAMPs) or pathogen-associated molecular patterns (PAMPs), leading to the recruitment and activation of Caspase-1. The activation of Caspase-1 facilitates the cleavage of pro-interleukin (IL)-1β and pro-IL-18, resulting in the maturation and release of IL-1β and IL-18. This process leads to cellular inflammation, pyroptosis, and an active participation in intrinsic immune defense [5]. Commonly identified inflammasomes include the NOD-like receptor pyrin domain-containing protein (NLRP) 1, NLRP2, NLRP3, NLRC4, NLRP6, NLRP12, absent in melanoma 2 (AIM2), and interferon-inducible protein 16 (IFI16) of the pyrin and hematopoietic interferon-inducible nuclear (HIN) domain protein family. Among these, the structure and function of the NLRP3 inflammasome are comparatively well understood [6–8]. NLRP3 inflammasomes were activated in a high glucose (HG)-induced renal mesangial cell model. The long non-coding RNA (lncRNA) nuclear-enriched abundant transcript 1 (NEAT1) and its target gene microRNA (miRNA)-34c were identified as regulators of inflammation and pyroptosis in diabetic kidney disease (DKD), mediated through the activation of the NLRP3 inflammasome [9] This suggests that the inhibition of NLRP3 inflammasomes may offer a therapeutic target for inflammation-related diseases. Ubiquitination and deubiquitination modifications are essential types of protein post-translational modification (PTM), playing crucial regulatory roles in inflammatory signaling [10]. It has been reported that these modifications can directly or indirectly regulate the activation or degradation of NLRP3 inflammasomes, thereby intervening in the development of various inflammation-related diseases [11, 12]. Therefore, this review discusses the regulation of NLRP3 inflammasome activation by ubiquitination and deubiquitination modifications, their intervention in inflammation-related diseases, and the potential mechanisms associated with these processes. This study aims to serve as a theoretical reference for future research on the ubiquitination and deubiquitination modifications of NLRP3 inflammasomes in the treatment of inflammation-related diseases.

## Overview of NLRP3 inflammasomes

### NLRP3 inflammasome composition, structure, and function

The concept of NLRP3 inflammasomes was first proposed by Martinon et al. in 2002 [13], who reported that these NLRP3 inflammasomes are widely distributed in various cells, including lymphocytes, microglia, neutrophils, and neuronal cells. NLRP3 inflammasomes can recognize and respond to various physiological and pathological stimuli [14], positioning them as critical components of the innate immune system. These multimolecular complexes consist of the “sensor” NLRP3 protein, the “adapter” ASC, and the “effector” pro-Caspase-1 [15]. As the recognition protein of NLRP3 inflammasomes, the NLRP3 protein mainly contains three structural domains: the leucine-rich repeat (LRR) domain, the nucleotide-binding oligomerization (NACHT) domain, and the pyrin (PYD) domain [16]. The LRR domain is responsible for detecting and recognizing specific ligands and activating apical molecular mechanisms through ubiquitination modifications. It also regulates the related ubiquitin ligases, thus modulating the NLRP3 inflammasome initiation and mediating self-regulation and protein–protein interactions [17]. Aside from exerting specialized effects on the self-association and oligomerization of the NLRP3 protein, the NACHT domain also produces an active response to adenosine triphosphate (ATP) enzymes and promotes the activation of apoptotic protease-activating factor [18]. However, under normal conditions, the LRR structural domain can affect or inhibit the recruitment of NACHT, thereby inhibiting the NLRP3 inflammasome generation [19]. The PYD domain primarily recruits downstream effector signaling molecules. The ASC, an articulating protein, possesses two structural domains: a PYD at the C-terminus that binds to NLRP3 and a CARD at the N-terminus that binds to pro-Caspase-1, which is involved in the production of inflammasomes [20]. Upon stimulation, the effector protein pro-Caspase-1, which mainly consists of CARD, undergoes cleavage to produce Caspase-1. This process initiates an inflammatory cascade response and pyroptosis within the NLRP3 pathway [21, 22].

### Mechanisms of NLRP3 inflammasome activation

**Figure 1.**
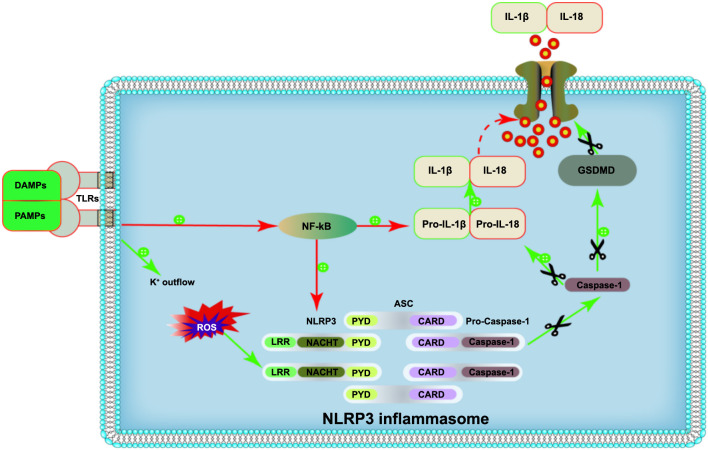
**Mechanism of activation of the NLRP3 inflammasome.** NLRP3: NOD-like receptor pyrin-domain containing protein; DAMPs: Damage-associated molecular patterns molecules; PAMPs: Pathogen-associated molecular patterns molecules; TLRs: Toll-like receptors; ROS: Reactive oxygen species; NF-κB: Nuclear factor-kappa B; LRR: Leucine-rich repeat domain; NACHT: Nucleotide binding oligomerization domain; PYD: Pyrin domain; ASC: Apoptosis-associated speck-like protein; CARD: Caspase activation and recruitment domain; Pro-Caspase-1: Pro-cysteinyl aspartate specific proteinase-1; IL: Interleukin; GSDMD: Gasdermin D.

When an abnormal internal or external stimulus triggers an immune response, the NLRP3 inflammasomes are immediately assembled and activated [23, 24]. The activation mechanism of NLRP3 inflammasomes involves two stages, the initiation signaling and the activation signaling. The initiation signaling involves the activation of PRRs, such as toll-like receptors (TLRs) and tumor necrosis factor-alpha (TNF-α) by PAMPs and DAMPs. This activation subsequently promotes the nuclear factor-kappa B (NF-κB) activation, which in turn upregulates the expression of pro-Caspase-1, pro-IL-1β, and pro-IL-18 [25]. The activation signaling occurs after the initiating signal is sent. PAMPs and DAMPs induce abnormal body functions (such as K^+^ efflux, Cl^-^ efflux, Ca^2+^ flow, Na^+^ influx, mitochondrial damage, Golgi catabolism, and lysosomal cleavage). These changes further induce NLRP3 inflammasome activation [26]. Upon stimulation, common ASC-containing adaptor molecules are recruited to promote the formation of the NLRP3-ASC complex [27]. The exposed CARD interconnects with the CARD portion of the inactive pro-Caspase-1. This interaction leads to the generation of active Caspase-1 following the cleavage of pro-Caspase-1. The activated Caspase-1 then cleaves pro-IL-1β and pro-IL-18, ultimately resulting in the production of mature and biologically active IL-1β and IL-18, thereby inducing an inflammatory cascade response [21]. Furthermore, the activated Caspase-1 cleaves the gasdermin D protein (GSDMD), releasing its functional N-terminus, which translocates to the plasma membrane to form a pore. This process enables the release of mature IL-1β and IL-18 into the extracellular compartment and mediates cytosolic rupture, leading to pyroptosis [28] (Figure 1).

### Ubiquitination/deubiquitination post-translational modification (PTM) mechanisms

Protein PTMs tightly regulate the activation or degradation of NLRP3 inflammasomes, including phosphorylation, ubiquitination, sumoylation, acetylation, ADP-ribosylation, S-nitrosylation, and palmitoylation. Among them, ubiquitination and deubiquitination modifications are common multifunctional protein PTMs that control the inflammatory immune response by regulating the composition of many NLRP3 inflammasomes and have emerged as essential mechanisms in regulating NLRP3 inflammasome signaling [29, 30]. In cells, the ubiquitin (Ub)-proteasome system (UPS), also known as the Ub-proteasome pathway (UPP), mediates the specific modification of target proteins by Ub under both pathological and physiological conditions. The UPS consists of six main components: Ub, Ub-activating enzymes (E1s), Ub-conjugating enzymes (E2s), E3 Ub ligase, 26S proteasome, and deubiquitinating enzymes (DUBs) [31]. Ub, first reported by Goldstein et al. in 1975, is a small signaling protein composed of 76 amino acids. It binds to substrate proteins via isopeptide bonds and is a critical messenger molecule for intracellular degradation and regulation of protein function [32]. Ubiquitination is the process of specific recognition and modification of target proteins by Ub, facilitated by the actions of E1s, E2s, and E3 Ub ligases, which exert biological effects, such as intracellular protein localization, metabolism, functional activity regulation, and protein degradation [33]. The ubiquitination cascade can be divided into three steps. Step one is the formation of a thioester bond between the cysteine residue of E1s and the C-terminal lysine residue of Ub, activating the Ub. Step two is the coupling of the E1s-activated Ub to the cysteine residue of E2s. Finally, step three encompasses the transfer of the activated Ub, bound to the E2s, to the substrate protein via E3 Ub ligase [34]. The Ub comprises seven lysine (K) residues, one N-terminal methionine (Met1) residue, and one C-terminal glycine (Gly) residue (K6, K11, K27, K29, K33, K48, K63, Met1, and Gly) [31]. Ubiquitination can be classified into three types, based on the number of Ub connections, polyubiquitin chain sites, and attachment types after ubiquitination: monoubiquitination (when ubiquitination occurs at a single residue), multimeric ubiquitination (when ubiquitination occurs at different sites of the same protein), and polyubiquitination (when multiple ubiquitinations occur at the same site) [35]. Ubiquitination modification is reversible, with DUBs capable of removing covalent bonds between Ub and the substrate, as well as between Ub molecules. This promotes the detachment of Ub from ubiquitinated protein substrates or proteasomal degradation substrates, allowing them to re-enter the intracellular protein cycle. In return, this reverses the effects of ubiquitination modifications and thus regulates the biological processes of the substrate [36]. DUBs are categorized into five main types based on their catalytic structural domains: Ub-specific proteases (USPs)/Ub-binding proteins (UBPs), Ub C-terminal hydrolases (UCHs), JAMM domain proteins (JAMMs), ovarian tumor proteases (OTUs), and Machado–Joseph domain proteases (MJDs) [37].

## Ubiquitination/deubiquitination regulates NLRP3 inflammasomes

### Ubiquitination regulates NLRP3 inflammasomes

Various enzymes catalyze the ubiquitination modification of NLRP3 inflammasomes (Table 1). A study has found that E3 Ub ligases target NLRP3 proteins or their constituents to degrade NLRP3 inflammasomes. The tripartite motif-containing proteins (TRIMs) family, known for its E3 Ub ligase activity, plays a significant role in this process. Specifically, TRIM24 has been shown to promote NLRP3 inflammasome ubiquitination in patients with endometriosis. This downregulates NLRP3 protein expression and inhibits inflammation, pyroptosis, and migration of endometrial mesenchymal cells [38]. In the context of chronic gastritis plus *Helicobacter pylori* (HP) infection in mice, and in an in vitro HP-induced gastric mucosal epithelial cell (GES-1) model, TRIM31 was found to directly bind to NLRP3 inflammasomes. This binding promoted the ubiquitination and subsequent degradation of NLRP3 inflammasomes, inhibiting mitochondrial damage, reducing mitochondrial reactive oxygen species (ROS) production, promoting GES-1 autophagy, and inhibiting lysosomal degradation. As a result, this slows down the GES-1 inflammatory response to HP infection [39]. In addition, in an in vitro IgA nephropathy model using immunoglobulin A1 (IgA1)-induced glomerular mesangial cells (GMCs), TRIM40 was observed to interact directly with NLRP3 inflammasomes. This interaction mediated the ubiquitination and degradation of NLRP3 inflammasomes, leading to reduced NLRP3 protein expression and consequently suppressing GMCs inflammation and proliferation [40]. In a lipopolysaccharide (LPS)-induced production in vitro model of mouse bone marrow-derived macrophages (BMDMs), BTB/POZ domain-containing protein 2 (BPOZ-2), possessing E3 Ub ligase activity, promoted ubiquitination modification of NLRP3 inflammasomes by recruiting cullin 3 (CUL3). This process downregulated the expression of NLRP3 inflammasomes and IL-1β proteins, thereby inhibiting the inflammatory response in BMDMs [41]. In mammary tissues of mastitis-affected cows and mammary tissues of mice injected with *Streptococcus lactis*, a notable reduction in the expression of cyclin-dependent kinase 5 (CDK5) regulatory subunit-associated protein 3 (CDK5RAP3) was observed, and such reduction led to the enhancement of NLRP3 and IL1β protein expression and aggravation of autophagy and apoptosis. However, CDK5RAP3 overexpression in mouse mammary tissues promoted its specific binding to NLRP3 inflammasomes, inducing ubiquitination modification of NLRP3 inflammasomes, facilitating the inactivation of NLRP3 inflammasomes, downregulating the expression of proteins downstream of NLRP3 inflammasomes, and thus inhibiting inflammation and pyroptosis in mastitis cells [42].

**Table 1:** Regulation of the NLRP3 inflammasome by ubiquitination/deubiquitination

**Enzymes/Proteins**	**PTM type**	**NLRP3 expression**	**Reference**
TRIM24*↑*	Ubiquitination*↑*	*↓*	[38]
TRIM31*↑*	Ubiquitination*↑*	*↓*	[39]
TRIM40*↑*	Ubiquitination*↑*	*↓*	[40]
BPOZ-2*↑*	Ubiquitination*↑*	*↓*	[41]
CDK5RAP3*↓*	Ubiquitination*↑*	*↓*	[42]
HUWE1*↑*	Ubiquitination*↑*	*↑*	[43]
TRIM62*↑*	Ubiquitination*↑*	*↑*	[44]
Ubc13*↑*	Ubiquitination*↑*	*↑*	[45]
USP16*↑*	Deubiquitination*↑*	*↑*	[46]
OTUD6A*↑*	Deubiquitination*↑*	*↑*	[47]
USP9X*↑*	Deubiquitination*↑*	*↑*	[48]
USP14*↑*	Deubiquitination*↑*	*↑*	[49]
UCHL5*↓*	Deubiquitination*↓*	*↓*	[50]
USP47*↓*	Deubiquitination*↓*	*↓*	[51]
STAMBP*↑*	Deubiquitination*↑*	*↓*	[52]
BRCC3*↓*	Deubiquitination*↓*	*↓*	[53]
USP30*↓*	Deubiquitination*↓*	*↓*	[54]

*↑* indicates upregulated, while *↓* indicates downregulated. NLRP3: NOD-like receptor pyrin-domain containing protein; PTM: Post-translational modification; TRIMs: Tripartite motif-containing proteins; BPOZ-2: Blood POZ containing gene type 2; CDK5RAP3: CDK5 regulatory subunit-associated protein 3; CDK5: Cyclin-dependent kinase 5; HUWE1: HECT, UBA, and WWE domain containing 1; Ubc13: Ubiquitin-conjugating enzyme E2 N; USPs: Ubiquitin specific proteases; OTUD6A: OTU domain-containing protein 6A; UCHL5: Ubiquitin carboxyl-terminal hydrolase L5; STAMBP: STAM-binding protein; BRCC3: BRCA1/BRCA2-containing complex subunit 3.

Several studies have highlighted the role of E3 Ub ligase in activating NLRP3 inflammasomes and upregulating their expression. In mouse BMDMs induced with LPS and ATP, the homologous to the E6-AP carboxyl terminus (HECT), Ub-associated domain (UBA), and WWE domain-containing E3 Ub protein ligase 1 (HUWE1) was found to interact with the NACHT structural domain of NLRP3 proteins through its Bcl-2 homology 3 (BH3) structural domain. This interaction induced k27-linked polyubiquitination of NLRP3 proteins, facilitating the activation of the NLRP3 inflammasomes and thereby increasing the expression of downstream proteins and the maturation of Caspase-1. Consequently, this process aggravated the inflammatory response in LPS- and ATP-induced murine BMDMs [43]. TRIM62 was significantly upregulated in oxygen–glucose deprivation (OGD)-treated microglia and mouse brain ischemia-reperfusion injury (I/R) models. The knockdown of TRIM62 in the I/R model suppressed the neuroinflammatory response, likely due to TRIM62 associating with NLRP3 inflammasomes in OGD-treated microglia. This association involved k63-linked polyubiquitylation, leading to a significant upregulation of NLRP3 protein expression and promoting microglial inflammatory responses [44]. Moreover, in LPS- and ATP-induced mouse BMDMs, the E2 Ub-conjugating enzyme E2 N (Ubc13) was identified to interact with NLRP3 inflammasomes. This interaction led to the induction of k63-conjugated polyubiquitination of NLRP3 inflammasomes, resulting in an increased expression of NLRP3 and IL-1β proteins, thereby exacerbating the inflammatory response in BMDMs [45].

### Deubiquitination regulates NLRP3 inflammasome activation

Deubiquitination modifications regulate NLRP3 inflammasome activation through numerous DUBs (Table 1). In a monosodium urate-induced gouty arthritis cell model, the USP16 DUB directly interacted with and mediated the deubiquitination of dynamin-related protein 1 (Drp1). This interaction led to an upregulation of Drp1 expression, promoted the activation of NLRP3 inflammasomes, and exacerbated the progression of gouty arthritis [46]. In an in vitro cellular model of dextran sulfate sodium (DSS)-induced colitis, the DUB OTU domain-containing protein 6A (OTUD6A) interacted with structural domains of the NLRP3 inflammasomes inducing their deubiquitination. This process enhanced the stability of the NLRP3 inflammasome, increased the expression of its relevant component proteins, and facilitated the DSS-induced inflammatory response [47]. Additionally, in an in vitro model using LPS-induced human lung epithelial cells, the USP9X DUB induced deubiquitination modification of NLRP3 inflammasomes, which activated NLRP3 inflammasomes, induced IL-1β and IL-18 maturation, and enhanced LPS-induced pyroptosis and inflammation [48]. Furthermore, in the annulus fibrosus cells derived from patients with intervertebral disc degeneration (IDD), overexpression of the USP14 DUB promoted the deubiquitination of NLRP3 inflammasomes. This resulted in the upregulation of NLRP3 protein expression and promoted pyroptosis in annulus fibrosus cells. Conversely, the downregulation of USP14 expression led to the degradation of NLRP3 inflammasomes and inhibited pyroptosis in these cells [49].

Deubiquitination modifications play a pivotal role in mediating the inhibition of NLRP3 inflammasome activation by DUBs. In chronic hepatitis C virus (HCV)-infected hepatocytes, the application of a chemical inhibitor of DUBs (WP1130) or the knockdown of Ub carboxyl-terminal hydrolases L5 (UCHL5) prevented the deubiquitination modification of the NLRP3 inflammasomes. This intervention downregulated the expression of NLRP3 inflammasomes and inhibited the inflammatory response to HCV infection [50]. The silencing of lncRNA ZNF883 in an epileptic neuronal cell model inhibited the molecular sponging effect on miR-138-5p, which in turn downregulated the USP47 DUB expression. This downregulation of DUB USP47 expression led to the inhibition of deubiquitination modification of NLRP3 inflammasomes, degradation of NLRP3 inflammasomes, reduced expression of TNF-α and NLRP3 proteins, and subsequently suppressed neuronal cell inflammation and apoptosis [51]. In a study involving an LPS-induced human monocyte-macrophage model, STAM-binding protein (STAMBP) DUB cleaved the k63-linked Ub chain of NLRP3 inflammasomes. This cleavage induced deubiquitination modification of NLRP3 inflammasomes, suppressed their expression, and consequently prevented inflammation [52]. Additionally, lentivirus-mediated overexpression of E3 Ub ligase WWP2 in LPS- and Nigerian mycobacteria-induced mouse BMDMs reduced BRCA1/BRCA2-containing complex subunit 3 (BRCC3) protein levels in DUBs. This reduction inhibited the deubiquitylation modification of NLRP3 inflammasomes, leading to their degradation and consequently inhibiting the inflammatory response [53]. In another study, the knockdown of DUB USP30 in advanced glycation end products (AGEs)-treated human skin fibroblast model inhibited the NLRP3 inflammasome deubiquitination. This inhibition led to the degradation of NLRP3 inflammasomes, blocked NLRP3 inflammasome signaling, and enhanced human skin fibroblast viability [54].

## NLRP3 inflammasome ubiquitination/deubiquitination and inflammation-related diseases

Inflammation serves as the body’s defense mechanism against tissue damage, infection, or other abnormal stimuli. The inflammatory response facilitates the removal of pathogens, repairs tissue damage, and restores normal tissue function [55]. The NLRP3 inflammasome is a crucial factor in inducing the inflammatory cascade. Excessive activation of the NLRP3 inflammasome can lead to sustained or excessive inflammatory responses, contributing to the pathogenesis of various inflammation-related diseases [56]. Increasing evidence suggests that ubiquitination and deubiquitination modifications are important mechanisms in regulating NLRP3 inflammasome activation. These modifications represent a potential strategy for the prevention and treatment of inflammation-related diseases (Table 2).

**Table 2:** The roles of ubiquitination/deubiquitination of NLRP3 inflammasome in inflammation-related diseases

**Inflammation-related diseases**	**Enzymes/ Compounds**	**PTM type**	**NLRP3 expression**	**Main functions**	**Reference**
Diabetic kidney disease	SPOP *↑*	Ubiquitination *↑*	*↓*	Inhibition of inflammatory response and alleviation of podocyte injury	[59]
Diabetic cardiomyopathy	Spd *↑*	Ubiquitination *↓*	*↓*	Reduction of collagen secretion from cardiac fibroblasts, and significant amelioration of HG-induced fibroblast proliferation, migration, inflammation, and pyroptosis	[60]
Diabetic retinopathy	SYVN1 *↑* BRCC3 *↓*	Ubiquitination *↑*	*↓*	Reduction of HG-induced inflammatory cytokine secretion from Müller cells, and alleviation of Müller cell injury	[61]
Diabetes mellitus	HECTD3 *↑*	——	*↑*	Promotion of neural precursor pyroptosis, impairment of diabetic rats learning and memory functions	[62]
Parkinson’s disease	MIB1 *↓*	Ubiquitination *↓*	*↑*	Promotion of SH-SY5Y inflammatory response and pyroptosis	[65]
Parkinson’s disease	Parkin *↓*	Ubiquitination *↓*	*↑*	Exacerbation of microglia neuroinflammation and loss of dopaminergic neurons	[66]
Parkinson’s disease	Ka *↑*	Ubiquitination *↑*	*↓*	Slowing down neuroinflammation and neuronal damage	[67]
Alzheimer disease	NEDD4 *↓*	——	*↓*	Inhibition of the hippocampal inflammatory response, improvement of working and spatial memory deficits in AD mice	[68]
Osteoarthritis	LKB1 *↑*	Ubiquitination *↑*	*↓*	Inhibition of OA chondrocyte inflammation and pyroptosis	[71]
Osteoporosis	USP1 *↑*	Ubiquitination *↓*	*↓*	Suppression of H_2_O_2_-induced osteoblast inflammation and pyroptosis	[72]
Osteoarthritis	USP7 *↑*	Ubiquitination *↓*	*↑*	Exacerbation of pyroptosis and extracellular matrix remodeling in OA chondrocytes	[73]
Intervertebral disc degeneration	Platelet-rich plasma-derived exosomes *↑*	Ubiquitination *↑*	*↓*	Significant inhibition of inflammatory responses and apoptosis in intervertebral discs of rats with IDD	[74]
Acute colitis	BRCC3 and JOSD2 *↓*	Deubiquitination *↓*	*↑*	Suppression of IL-1β and IL-18 maturation and pyroptosis levels, and amelioration of acute colitis	[76]
Colitis-associated colorectal cancer	Cullin1 *↑*	Ubiquitination *↑*	*↓*	Inhibition of the expression of NLRP3, IL-6, and TNF-α, and prevention of CAC	[77]
Acute colitis	CA *↑*	Ubiquitination *↓*	*↓*	Reduction of the colonic inflammatory response and oxidative stress, and prevention of the development of acute colitis in mice	[78]
Ulcerative colitis	RNF183 *↓*	Ubiquitination *↓*	*↓*	Inhibition of colonic inflammation and oxidative stress, and alleviation of acute colitis lesions in mice	[79]

*↑* indicates upregulated, while *↓* indicates downregulated. —— represents no reports. NLRP3: NOD-like receptor pyrin-domain containing protein; PTM: Post-translational modification; SPOP: Speckle type BTB/POZ protein; Spd: Spermidin; HG: High glucose; SYVN1: Synoviolin 1; BRCC3: BRCA1/BRCA 2-containing complex subunit3; HECTD3: Homologous to the E6-associated protein carboxyl terminus domain containing 3; MIB1: Mindbomb E3 ubiquitin protein ligase 1; Ka: Kaempferol; NEDD4: Neural precursor cell expressed developmentally down-regulated protein 4; AD: Alzheimer disease; LKB1: Liver kinase B1; OA: Osteoarthritis; USPs: Ubiquitin specific proteases; IDD: Intervertebral disc degeneration; JOSD2: Josephin domain containing 2; IL: Interleukin; TNF-α: Tumor necrosis factor alpha; CAC: Colitis-associated colorectal cancer; CA: Carnosic acid; RNF183: Ring finger protein 183.

### NLRP3 inflammasome ubiquitination/deubiquitination and diabetic complications

Diabetes mellitus is a chronic endocrine disease caused by lipid metabolism disorders or insulin dysfunction. Persistent elevation of blood glucose levels can cause extensive vascular damage, leading to complications such as DKD, diabetic foot ulcers, diabetic retinopathy, and diabetic neuropathy [57]. In the HG state, the activation of several inflammatory cells and the release of inflammatory mediators contribute to vascular endothelial cell necrosis and reduced insulin sensitivity, key factors in the development of various diabetes-related complications [58]. Studies have confirmed that ubiquitination or deubiquitination modifications can inhibit the NLRP3 inflammasome activation, thereby slowing the pathophysiological processes of diabetic complications. A study observed a significant downregulation of speckle-type BTB/POZ protein (SPOP) in renal tissue samples from DKD patients. In streptozotocin (STZ)-induced DKD mice, the *Spop* gene deletion accelerated renal dysfunction and glomerular pathological changes. Further experiments revealed that SPOP promotes the degradation of the NLRP3 inflammasome by accelerating the polyubiquitination of k48-linked NLRP3 inflammasomes. This action inhibits the inflammatory response and alleviates podocyte injury in models of podocyte injury treated with HG [59]. Another study demonstrated that intraperitoneal injection of spermidine (Spd) significantly downregulated NLRP3 messenger RNA (mRNA) expression, inhibited collagen accumulation, and ameliorated myocardial tissue injury in the hearts of diabetic mice with myocardial injury. Cellular experiments have revealed that Spd in HG-induced mouse cardiac fibroblasts significantly inhibited transforming growth factor-β1 (TGF-β1) and phosphorylated phospho-Smad2 (p-Smad-2)/p-Smad-3 protein expression. It also inhibited the ubiquitylated degradation of Smad-7, thereby increasing Smad-7 expression, downregulating α-smooth muscle actin (α-SMA), collagen I (COL-I)/COL-III, matrix metalloproteinase 2 (MMP-2)/MMP-9, NLRP3, and GSDMD-N protein levels, and decreasing collagen secretion. These processes significantly ameliorated the proliferation, migration, inflammation, and pyroptosis of HG-induced fibroblasts [60].

Several studies have confirmed that ubiquitination and deubiquitination modifications promote the activation of the NLRP3 inflammasome and contribute to the exacerbation of injuries in diabetic complications. A notable finding was the decreased synoviolin 1 (SYVN1) expression in retinal tissues from HG-induced Müller cell models and STZ-induced diabetic retinopathy mouse models. Concurrently, an increase in BRCC3 expression was observed in DUBs, along with elevated levels of TNF-α, IL-1β, and IL-6. The cellular experiments revealed that the SYVN1-mediated downregulation of BRCC3 promotes ubiquitination modification of NLRP3 inflammasomes. This modification decreases NLRP3 protein levels and reduces the secretion of inflammatory cytokines from HG-induced Müller cells, thereby ameliorating Müller cell injury [61]. In the hippocampus of STZ-induced diabetic rats and in neural precursor cells (PC12) subjected to HG conditions, a significant increase in the levels of the E3 Ub-protein ligase HECTD3 was observed. The overexpression of HECTD3 was found to accelerate neuronal pyroptosis in the hippocampus of the rats. This overexpression also upregulated the expression of pro-inflammatory cytokines, including IL-1β, TNF-α, and IL-6 in the hippocampus, impairing brain function. Additionally, HECTD3 significantly promoted the protein expression of NLRP3, GSDMD, and IL-1β in HG-induced cell models, thereby facilitating pyroptosis [62].

In summary, the ubiquitination and deubiquitination modifications play a crucial role in the development of diabetic complications by regulating the NLRP3 inflammasome expression. However, the specific mechanisms underlying the ubiquitination and deubiquitination of the NLRP3 inflammasome in the context of diabetic complications remain not fully elucidated. Additionally, while the modification of the NLRP3 inflammasome through ubiquitination and deubiquitination is mainly mediated by E3 Ub ligase and DUBs, the roles of E1s or E2s in these processes have not been extensively reported, thus requiring further studies to fully understand these regulatory mechanisms.

### NLRP3 inflammasome ubiquitination/deubiquitination and neurodegenerative diseases (NDDs)

NDDs are characterized by the loss of neurons or nerve sheath cells [63]. Brain damage or infection rapidly activates microglia, leading to neurodegeneration. This process results in excessive infiltration of inflammatory mediators in the brain, causing neuronal dysfunction, oligodendrocyte necrosis, and demyelination damage [64]. Studies have confirmed that ubiquitination or deubiquitination modifications catalyze the maturation of NLRP3 inflammasomes and accelerate nerve injury. In an in vitro model using 1-methyl-4-phenylpyridinium (MPP^+^)-treated human neuroblastoma cells (SH-SY5Y), a significant downregulation of lncRNA zinc finger antisense 1 (ZFAS1) and an increase in miR-590-3p expression were observed. This altered expression inhibited mindbomb E3 Ub protein ligase 1 (MIB1) protein expression and directly interfered with the MIB1-dependent ubiquitination of thioredoxin interacting protein (TXNIP). As a result, NLPR3 inflammasomes were activated, promoting inflammatory response and pyroptosis in SH-SY5Y cells [65]. A loss of Parkin activity and overaccumulation of NLRP3 inflammasome was observed in the brain tissues of LPS-induced mouse models of Parkinson’s disease (PD). This phenomenon subsequently led to the development of cerebral impairment and dopaminergic neuron loss in the mice. Cellular experiments further confirmed that Parkin downregulation in LPS-induced primary microglia and SH-SY5Y cell models blocked k3-linked polyubiquitination of NLRP3 inflammasomes. This blockage promoted the NLRP3 inflammasome activation and exacerbated microglial neuroinflammation [66].

Research has established that ubiquitination and deubiquitination modifications play a significant role in degrading NLRP3 inflammasomes and inhibiting neuroinflammation. In models of PD, specifically dopaminergic neuronal cells induced by 1-methyl-4-phenyl-1,2,3,6-tetrahydropyridine (MPTP) and LPS-induced PD mouse brains, increased expression of NLRP1 and NLRP3 inflammasomes was observed. Administration of kaempferol (Ka) was found to attenuate the inflammatory response and neurodegeneration in these models. Particularly in the LPS-induced PD mouse brain model, Ka promoted the ubiquitination modification of the NLRP3 protein, thereby preventing its activation, reducing NLRP3 inflammasome expression, and slowing neuroinflammation and neuronal damage caused by overproduction of IL-1β [67]. In the context of Alzheimer’s disease (AD), studies using triple-transgenic mutant AD mouse models and wild mice treated with an E3 Ub ligase inhibitor, M01, demonstrated significant improvements in memory deficits, exploring the familiar and new arms of the T maze to the same extent. M01-treated AD mice showed a significantly shortened time and path length to reach a new location in the Morris water maze, indicating improved working and spatial memory. M01 administration led to a decrease in the expression of E3 Ub ligase neural precursor cell expressed, developmentally downregulated protein 4 (NEDD4), which has a high affinity for M01, in the hippocampus of the triple-transgenic mutant AD mice. Additionally, M01 promoted the expression of autophagy protein, inhibited the expression of NLRP3 inflammasomes and Caspase-1, and consequently inhibited the hippocampal inflammatory response of these mice [68].

In summary, both in vitro and in vivo experiments have established that NLRP3 inflammasomes can accumulate excessively in damaged brain tissues and neurons. Additionally, NLRP3 inflammasome ubiquitination and deubiquitination modifications have been shown to either inhibit or promote neuroinflammatory injuries. These modifications play a significant role in the pathological development of NDDs. However, the potential mechanisms through which the PTMs of NLRP3 inflammasomes can be leveraged for treating NDDs require further exploration. Moreover, while chemical inhibitors are known to directly or indirectly promote the ubiquitination or deubiquitination of NLRP3 inflammasomes, the detailed mechanisms underlying these effects are not yet thoroughly understood and warrant additional research.

### NLRP3 inflammasome ubiquitination/deubiquitination and musculoskeletal system diseases

The most prevalent musculoskeletal diseases include osteoarthritis (OA), osteoporosis (OP), and IDD. They are characterized by abnormalities in muscle and bone metabolism and various comorbidities, posing significant health risks to the elderly population [69]. Persistent low-grade joint inflammation is the leading cause of musculoskeletal diseases, where cartilage destruction results in the fragmentation of various extracellular matrices, triggering an inflammatory response in the joint cavity [70]. A study has reported that ubiquitination or deubiquitination modifications indirectly inhibit NLRP3 inflammasome activation and reduce inflammatory responses in musculoskeletal system diseases. In a model of LPS-induced human chondrocyte inflammation, overexpression of hepatic kinase B1 (LKB1) significantly enhanced LKB1 ubiquitination. This upregulation led to an increase in AMP-activated protein kinase (AMPK) expression, subsequently reducing NLRP3 inflammasome and ASC expression. As a result, inflammation and pyroptosis in OA chondrocytes were inhibited, slowing down the progression of OA [71]. Furthermore, in an in vitro model of H_2_O_2_-treated osteoblasts representing OP, a significant reduction in DUB USP1 expression was observed. USP1 interacted with the tumor necrosis factor receptor-associated factor 6 (TRAF6) and inhibited its ubiquitination, leading to the stabilization of TRAF6. This interaction blocked the NF-κB signaling, inhibiting the expression of NLRP3 inflammasomes, GSDMD-N, and Caspase-1. Consequently, this process inhibited the H_2_O_2_-induced osteoblast inflammatory response and pyroptosis [72]. Additional evidence suggests that ubiquitination or deubiquitination modifications can exacerbate musculoskeletal diseases by promoting conductance in the NLRP3 inflammasome upstream pathway. In an OA chondrocyte injury model, DUB USP7 expression was upregulated in a dose-dependent manner in response to H_2_O_2_, enhancing ROS production and significantly inhibiting chondrocyte proliferation. Silencing of USP7 protected the chondrocytes from H_2_O_2_-induced cellular injury. Additionally, in vitro experiments revealed that the overexpression of USP7 inhibited the ubiquitination degradation of nicotinamide adenine dinucleotide phosphate reduced oxidase 4 (NOX4), leading to elevated ROS production, which in turn increased NLPR3 inflammasome expression, resulting in the release of mature IL-1β and IL-18. Consequently, this promoted pyroptosis and extracellular matrix remodeling in OA chondrocytes [73]. Furthermore, in LPS- and IL-4-induced rat models of BMDMs, platelet-rich plasma-derived exosomes significantly enhanced NLRP3 inflammasome ubiquitination modification. This modification inhibited NLRP3 inflammasome activation and upregulated the expression of autophagy-associated protein 5 (ATG5), ATG7, and P62, which accelerated NLRP3 inflammasome autophagy degradation. This process led to a decrease in IL-1β secretion and promoted macrophage polarization, thereby alleviating inflammatory injury in BMDMs. Platelet-rich plasma-derived exosomes also significantly inhibited IL-1β-induced B-cell lymphoma-2-associated X protein (Bax) and decreased Caspase-3 protein levels in rat medullary cells. These exosomes upregulated B-cell lymphoma-2 (Bcl-2) expression, consequently inhibiting the apoptosis of medullary cells. Moreover, further studies confirmed that the injection of platelet-rich plasma-derived exosomes significantly inhibited inflammatory responses and apoptosis in the intervertebral discs of rats with IDD [74].

In summary, ubiquitination or deubiquitination modifications can indirectly inhibit the expression of NLRP3 inflammasomes and their downstream proteins. These modifications also have the potential to alleviate musculoskeletal diseases by modulating inflammatory responses. However, there is currently a relative lack of literature focusing on the application of NLRP3 inflammasomes in the clinical treatment of musculoskeletal diseases, with future research needed to explore its mechanism of action.

### NLRP3 inflammasome ubiquitination/deubiquitination and inflammatory bowel disease (IBD)

IBD is a nonspecific complex disorder characterized by numerous complications and involves the gastrointestinal mucosa and mucosal musculature. Gastrointestinal inflammation is an important causative factor for the development and progression of IBD [75]. A study found that deubiquitination modifications inhibited the NLRP3 inflammasome activation and suppressed IBD-associated gastrointestinal inflammation. The NLRP3-R779C variant, when introduced into human THP-1 cells, induced NLRP3 inflammasome assembly and increased its expression. This resulted in the enhancement of the inflammatory response and pyroptosis. In a DSS-induced cell model of acute colitis, the DUBs BRCC3 and Josephin domain containing 2 (JOSD2) were significantly upregulated, promoting the deubiquitination modification of the NLRP3-R779C variant. This activity facilitated NLRP3 inflammasome activation and exacerbated the pathological deterioration in acute colitis. Conversely, the downregulation of either BRCC3 or JOSD2 significantly inhibited the deubiquitination of NLRP3-R779C, suppressing IL-1β and IL-18 maturation and pyroptosis, thereby ameliorating acute colitis [76]. Further evidence suggested that the caffeic acid phenethyl ester (CAPE) administration blocked the interaction between NLRP3 inflammasomes and DUB COP9 signalosome 5 (CSN5) in azoxymethane (AOM)/DSS-induced colitis-associated colorectal cancer (CAC) mouse colon tissues. This blockade promoted the mutual binding of cullin 1 (CUL1) and NLRP3 inflammasome, and induced a ubiquitinated degradation of NLRP3 inflammasome, which in turn inhibited the expression of NLRP3, IL-6, and TNF-α, and prevented CAC [77]. Furthermore, the administration of cumaric acid (CA) has shown significant therapeutic effects in DSS-treated colitis mice. It notably ameliorated symptoms, such as colon shortening, fecal bleeding, and weight loss. CA also reduced the phosphorylation levels of the inhibitor of nuclear factor-κB α (IκBα) and c-Jun N-terminal kinase (JNK) in the colon and prevented macrophage infiltration. Additionally, it inhibited the ubiquitination degradation of nuclear factor erythroid-2-related factor 2 (Nrf2) by preventing the interaction between CUL2 and Kelch-like ECH-associated protein 1 (Keap1). This inhibition led to the downregulation of the NLRP3 inflammasome and intrinsic nitric oxide synthase (iNOS) expression, further reducing colonic inflammation and oxidative stress (OS), and alleviating the lesions associated with acute colitis [78]. Continuous moxibustion treatment for 16 days at bilateral Tianshu points on the abdomen of rats with DSS-induced ulcerative colitis resulted in significant improvements. The Disease Activity Index (DAI) scores of the rats were significantly reduced, and the intact mucosal structure of the colon was restored. This improvement was associated with the targeted inhibition of the expression of E3 ubiquitin ligase ring finger protein 183 (RNF183). This inhibition led to a reduction in the ubiquitination degradation of IκBα by preventing its binding with RNF183. This resulted in reduced levels of NF-κB and p65 proteins, ultimately inhibiting the expression of the NLRP3 inflammasome and IL-1β. These effects collectively attenuated the inflammatory response of colonic tissues and alleviated colonic injury in rats with ulcerative colitis [79].

In summary, ubiquitination modifications may have the potential to improve gastrointestinal injury in IBD by blocking the NLRP3 inflammasome activation. Additionally, various compounds and physical therapy methods may indirectly suppress the expression of NLRP3 inflammasomes through either ubiquitination or deubiquitination modifications. However, the direct association between these modifications and NLRP3 inflammasomes, as well as their precise roles in the development and progression of IBD, has not been extensively reported.

## Potential mechanisms of NLRP3 inflammasome ubiquitination/ deubiquitination intervention in inflammation-related diseases

### NLRP3 inflammasome ubiquitination/deubiquitination and the inflammatory response and pyroptosis

**Figure 2.**
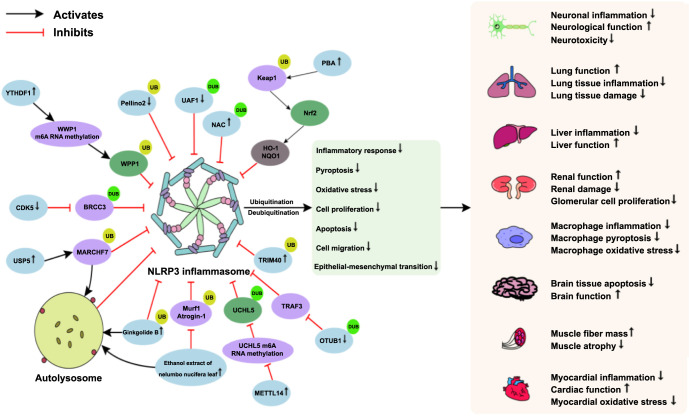
**Potential mechanisms of NLRP3 inflammasome ubiquitination or deubiquitination intervention in inflammation-related diseases.** NLRP3: NOD-like receptor pyrin-domain containing protein; DUBs: Deubiquitinating enzymes; UBs: Ubiquitinating enzymes; BRCC3: BRCA1/BRCA2-containing complex subunit3; CDK5: Cyclin dependent kinase 5; WWP1: WW domain-containing E3 ubiquitin protein ligase 1; YTHDF1: YTH domain family protein 1; m6A: N6-methyladenosine; USPs: Ubiquitin specific proteases; MARCHF7: Membrane-associated ring-CH-type finger 7; Murf 1: Muscle ring finger protein 1; Atrogin-1: Atrophy gene 1; UAF1: Ubiquitin specific peptidase 1-associated factor 1; NAC: N-acetyl-L-cysteine; PBA: Pubescenoside A; Keap1: Kelch-like ECH-associated protein 1; Nrf2: Nuclear factor erythroid-2-related factor 2; HO-1: Hemeoxygenase-1; NQO1: Nadph quinone oxidoreductase 1; METTL14: Methyltransferase-like 14; UCHL5: Ubiquitin carboxyl-terminal hydrolase L5; TRIMs: Tripartite motif-containing proteins; OTUB1: OTU domain containing ubiquitinaldehyde-binding protein 1; TRAF3: Tumor necrosis factor receptor associated factor 3.

NLRP3 inflammasome activation is a key factor in triggering inflammatory responses and pyroptosis, and the inhibition of its overactivation can help alleviate inflammation-related diseases [80]. In a study, the DUB BRCC3 was found to have significantly increased protein levels in mouse primary cortical neuron cells and in a mouse brain tissue model of PD, both induced by MPP^+^. Additionally, the overexpression of BRCC3 significantly exacerbated the inflammatory response of primary cortical neuron cells. Further cellular experiments confirmed an interaction between CDK5 and BRCC3. It was discovered that inhibiting CDK5 expression could reduce BRCC3 expression in MPP^+^-induced mouse primary cortical neuron cells. This inhibition blocked the interaction between BRCC3 and the NLRP3 inflammasome, thereby inhibiting the deubiquitination modification of NLRP3 inflammasomes. As a result, the maturation and release of NLRP3 inflammasomes and IL-1β were prevented, ultimately inhibiting the inflammatory response induced by MPP^+^ in mouse primary cortical neurons [81]. The expression of E3 Ub ligase WW domain-containing protein 1 (WWP1) and YTH domain family protein 1 (YTHDF1) was found to be downregulated in the serum of both LPS- and ATP-induced mouse macrophage models and in cecum ligation- and puncture-induced sepsis mouse models. The overexpression of YTHDF1 increased the N6-methyladenosine (m6A) RNA methylation level of WWP1. This methylation promoted the ubiquitination degradation of the NLRP3 inflammasome and downregulated the levels of TNF-α, IL-1β, and GSDMD proteins, leading to the inhibition of pyroptosis in mouse macrophages. Meanwhile, YTHDF1 overexpression also reduced inflammation in lung, liver, and kidney tissues in mice with sepsis and helped restore the damaged tissue structures [82]. In addition, Pellino2, a protein that can bind to NLRP3, was found to be involved in the regulation of the NLRP3 inflammasome in LPS- and ATP-induced THP-1 cell models. Overexpression of Pellino2 in a mycoplasma pneumonia (MPP) cell model promoted the activation of NLRP3 inflammasome ubiquitination. Conversely, the inhibition of Pellino2 expression resulted in reduced levels of TNF-α, interferon-γ (IFN-γ), and GSDMD protein expression in MPP mice and in vitro cellular models. This reduction attenuated lung tissue damage and inhibited inflammatory responses and pyroptosis [83]. In conclusion, ubiquitination and deubiquitination processes can effectively inhibit inflammatory cascade reactions and pyroptosis by regulating the expression of NLRP3 inflammasomes. This regulatory mechanism contributes to the improvement of inflammation-related diseases (Figure 2).

### NLRP3 inflammasome ubiquitination/deubiquitination and autophagy

Autophagy, a lysosome-mediated active cellular degradation process, selectively removes unwanted intracellular components, such as damaged organelles, pathogens, and invasive bacteria, ensuring normal cellular survival and function [84]. In LPS-induced mouse peritoneal macrophages, the DUB USP5 has been shown to directly bind to membrane-associated ring-CH-type finger 7 (MARCHF7). This interaction promotes k48-linked polyubiquitination degradation of NLRP3 inflammasomes. In addition, USP5 mediates the autophagy–lysosomal pathway to promote NLRP3 inflammasome degradation. Consequently, this leads to the downregulation of IL-1β, IL-6, and TNF-α protein levels, ultimately inhibiting LPS-induced inflammatory responses [85]. In another study, ginkgolide B was found to induce the binding of NLRP3 inflammasomes to Ub in LPS- and ATP-induced mouse microglia. This promoted the ubiquitination degradation of NLRP3 inflammasomes and increased the expression of ATG5, ATG7, and microtubule-associated protein 1 light chain 3-II (LC3-II)/LC3-I. This process also downregulated the sequestosome 1 (SQSTM1) protein levels, leading to the inhibition of NLRP3, Caspase-1, and IL-1β expression. Moreover, it regulated the balance between the M1 and M2 phenotypes of microglia. Animal experiments further confirmed that ginkgolide B increased autophagy protein expression in the hippocampal tissues of AD mice. It facilitated the degradation of NLRP3 inflammasomes via the autophagy–lysosomal pathway and ubiquitination modification, leading to significant improvements in cognitive behavior in AD mice [86]. An ethanol extract of *Nelumbo nucifera* leaves has been found to significantly reduce muscle ring finger protein 1 (Murf1), atrophy gene 1 (Atrogin-1), B-cell lymphoma-2-interacting myosin-like coiled-coil protein 1 (Beclin1), LC3I/II, and P62 protein levels in muscle tissues of mice suffering from muscle atrophy. This reduction, in turn, decreased the levels of NLRP3, Caspase-1, IL-1β, and GSDMD proteins. Furthermore, the ethanol extract downregulated the expression of autophagy proteins, pyroptosis-associated proteins, and NF-κB in dexamethasone-treated C2C12 myoblasts. This suggests that the extract may degrade NLRP3 inflammasomes by blocking both the Beclin-1/LC3I/II-mediated autophagy pathway and the Murf1/Atrogin-1-mediated UPS pathways. Consequently, the extract appears to inhibit inflammatory responses and pyroptosis in C2C12 myofibroblasts, thereby alleviating muscle atrophy [87]. In conclusion, the enhancement of autophagy appears to synergize with ubiquitination modifications to degrade NLRP3 inflammasomes effectively, thereby ameliorating inflammatory injury (Figure 2).

### NLRP3 inflammasome ubiquitination/deubiquitination and oxidative stress

OS is a pathogenic mechanism characterized by an increased production of ROS or reactive nitrogen species (RNS), leading to a dysregulation of the body’s antioxidant system [88]. OS can create an inflammatory microenvironment that contributes to the development of inflammation-related diseases, thereby impairing normal tissue and organ function [89]. In a study examining postoperative cognitive dysfunction (POCD) in rats, it was found that levels of Ub-specific peptidase 1-associated factor 1 (UAF1), malondialdehyde (MDA), TNF-α, IL-1β, and IL-18 were significantly elevated in the hippocampus of rats induced with surgery and sevoflurane (SEV) anesthesia. Concurrently, levels of superoxide dismutase (SOD) and catalase (CAT), which are key antioxidants, were reduced. This imbalance contributed to enhanced apoptosis and cognitive impairment in the hippocampus of these rats. However, the silencing of UAF1 appeared to promote the SEV-induced ubiquitination and subsequent degradation of NLRP3 inflammasomes in the hippocampus. This led to a downregulation of hippocampal NLRP3, IκBα, p65, and MDA expression, and an upregulation of SOD and CAT protein levels. As a result, hippocampal inflammation and OS were inhibited, which in turn alleviated cognitive impairment and neurotoxicity in the rats [90]. In a mouse model of oxygen–glucose deprivation/reperfusion (OGD/R)-induced myocardial I/R, pubescenoside A (PBA) demonstrated selective and covalent binding to the conserved cysteine (Cys) residues Cys77 and Cys434 of Keap1. This binding inhibited the ubiquitination of Nrf2 and upregulated the protein levels of heme oxygenase-1 (HO-1) and NADPH quinone oxidoreductase 1 (NQO1). Consequently, this inhibited the maturation of NLRP3 inflammasomes, alleviated OGD/R-induced myocardial inflammation and OS, and slowed down myocardial injury. These findings were further confirmed through in vivo experiments [91]. In another study, cecal ligation puncture led to an increased release of IL-1β and neutrophil extracellular traps (NETs) in lung tissues of septic lung-injured rats, which was positively correlated with the severity of lung injury in these rats. In a mouse alveolar macrophage model induced by LPS and NETs, N-acetyl-L-cysteine (NAC) prevented NLRP3 inflammasomes from undergoing deubiquitination modification by binding to DUBs. This inactivated the NLRP3 inflammasomes, which led to a downregulation in the levels of NLRP3, Caspase-1, and GSDMD-N proteins and suppressed ROS generation. Consequently, this inhibited macrophage inflammation, pyroptosis, and OS. Moreover, intraperitoneal injection of NAC in septic lung-injured rats similarly attenuated inflammatory responses and pyroptosis in lung tissues [92]. In conclusion, the ubiquitination or deubiquitination modification of NLRP3 inflammasomes plays a crucial role in inhibiting OS and slowing the pathological progression of inflammation-related diseases (Figure 2).

### NLRP3 inflammasome ubiquitination/deubiquitination and proliferation

Proliferation is an essential mechanism for maintaining normal physiological functions and repairing damaged tissues. However, dysregulated proliferation can contribute to the progression of inflammation-related diseases [93]. In a study involving patients with atherosclerosis (AS), it was found that DUB UCHL5 levels were significantly elevated in their blood samples. Similar results were obtained using oxidized low-density lipoprotein (ox-LDL)-induced vascular smooth muscle cell (VSMC) models. However, the silencing of UCHL5 in mice with a high-fat diet-induced AS led to an amelioration of plaque deterioration. Additionally, UCHL5 silencing inhibited ox-LDL-induced VSMC proliferation, migration, and inflammatory response. The overexpression of methyltransferase-like 14 (METTL14) promoted the binding of YTHDF1 to UCHL5 mRNA, which in turn inhibited the m6A methylation level of UCHL5. This downregulation of UCHL5 expression prevented the deubiquitination modification of NLRP3 inflammasomes, leading to reduced levels of NLRP3, ASC, Caspase-1, and IL-1β proteins. As a result, the proliferation of VSMCs induced by ox-LDL was inhibited [94]. In an IgA nephropathy cell model established by treating human GMCs with IgA1, a study confirmed a significant increase in NLRP3, IL-1β, and IL-18 protein levels. This elevation promoted the overproliferation of GMCs. Additionally, the expression of TRIM40 was significantly downregulated in this model. However, overexpressing TRIM40 led to its interaction with NLRP3 inflammasomes, promoting the ubiquitination and subsequent degradation of NLRP3 inflammasomes. This downregulation of NLRP3 inflammasome protein levels resulted in inhibited proliferation of GMCs and a slowdown in kidney injury progression [40]. The expression of DUB OTU domain-containing Ub aldehyde-binding protein 1 (OTUB1) was significantly elevated in a TGF-β1-induced human bronchial epithelial cell model and in the bronchial mucosal tissues of asthma patients. In this bronchial epithelial cell model, the knockdown of OTUB1 accelerated the ubiquitination degradation of TRAF3, leading to the inactivation of the NLRP3 inflammasome and downregulation of marker proteins such as NLRP3, GSDMD-N, α-SMA, COL-I, fibronectin-1, Bax, and E-cadherin. Consequently, this inhibited the bronchial epithelial cell inflammatory response, proliferation, apoptosis, migration, and epithelial–mesenchymal transition. However, overexpressing TRAF3 was found to be damaging to human bronchial epithelial cells [95]. In conclusion, the ubiquitination or deubiquitination of NLRP3 inflammasomes plays a critical role in inhibiting abnormal cell proliferation and plays a protective role against inflammation (Figure 2).

## Conclusion

The NLRP3 inflammasomes are vital mediators in the inflammatory cascade response, significantly impacting the progression of various inflammation-related diseases, such as diabetic complications, NDDs, musculoskeletal diseases, and IBD. Blocking the hyperactivation of NLRP3 inflammasomes may be a potential target for the prevention and treatment of inflammation-related diseases. Ubiquitination and deubiquitination modifications serve as key regulatory mechanisms in controlling the hyperactivation of NLRP3 inflammasomes. These PTMs can influence the activity and stability of NLRP3 inflammasomes, thereby exerting protective effects in inflammation-related diseases. By modulating the NLRP3 inflammasome pathway, these modifications address various pathological processes central to these diseases, such as excessive inflammatory responses, pyroptosis, abnormal autophagy, proliferation disorders, and OS damage.

In recent years, there has been a growing focus on understanding the role of ubiquitination and deubiquitination modifications in regulating NLRP3 inflammasome activation or degradation in inflammation-related diseases. Despite significant advancements, there remains a need for deeper exploration into the mechanisms of these modifications and the specific roles of NLRP3 inflammasomes in such diseases. This paper has delved into the regulation of NLRP3 inflammasome activation by ubiquitination and deubiquitination modifications, examining their roles in inflammation-related diseases and the underlying potential mechanisms. By providing a comprehensive review of current knowledge in this field, this paper aims to serve as a theoretical reference for future research efforts. These efforts are geared toward developing novel treatment strategies for inflammation-related diseases by targeting the ubiquitination or deubiquitination of NLRP3 inflammasomes.

## References

[1] Zhang K, Kaufman RJ (2008). From endoplasmic-reticulum stress to the inflammatory response. Nature.

[2] Oz HS (2017). Chronic inflammatory diseases and green tea polyphenols. Nutrients.

[3] Bando M (2023). Rethinking treatment strategies for idiopathic pulmonary fibrosis: reevaluation of anti-inflammatory and immunosuppressive therapies. Respir Investig.

[4] Xiong W, Meng XF, Zhang C (2020). Inflammasome activation in podocytes: a new mechanism of glomerular diseases. Inflamm Res.

[5] Chen S, Sun B (2013). Negative regulation of NLRP3 inflammasome signaling. Protein Cell.

[6] Guo Y, Hong W, Wang X, Zhang P, Körner H, Tu J (2019). MicroRNAs in microglia: How do microRNAs affect activation, inflammation, polarization of microglia and mediate the interaction between microglia and glioma?. Front Mol Neurosci.

[7] Velloso FJ, Trombetta-Lima M, Anschau V, Sogayar MC, Correa RG (2019). NOD-like receptors: major players (and targets) in the interface between innate immunity and cancer. Biosci Rep.

[8] Moltrasio C, Romagnuolo M, Marzano AV (2022). NLRP3 inflammasome and NLRP3-related autoinflammatory diseases: from cryopyrin function to targeted therapies. Front Immunol.

[9] Zhan JF, Huang HW, Huang C, Hu LL, Xu WW (2020). Long non-coding RNA NEAT1 regulates pyroptosis in diabetic nephropathy via mediating the miR-34c/NLRP3 axis. Kidney Blood Press Res.

[10] Cockram PE, Kist M, Prakash S, Chen SH, Wertz IE, Vucic D (2021). Ubiquitination in the regulation of inflammatory cell death and cancer. Cell Death Differ.

[11] Pu Z, Sui B, Wang X, Wang W, Li L, Xie H (2023). The effects and mechanisms of the anti-COVID-19 traditional Chinese medicine, dehydroandrographolide from Andrographis paniculata (Burm.f.) Wall, on acute lung injury by the inhibition of NLRP3-mediated pyroptosis. Phytomedicine.

[12] Kim JS, Kim HK, Lee J, Jang S, Cho E, Mun SJ (2023). Inhibition of CD82 improves colitis by increasing NLRP3 deubiquitination by BRCC3. Cell Mol Immunol.

[13] Martinon F, Burns K, Tschopp J (2002). The inflammasome: a molecular platform triggering activation of inflammatory caspases and processing of proIL-beta. Mol Cell.

[14] Du X, Que W, Hu X, Yu X, Guo WZ, Zhang S (2021). Oridonin prolongs the survival of mouse cardiac allografts by attenuating the NF-κB/NLRP3 pathway. Front Immunol.

[15] Shahi A, Afzali S, Amirzargar A, Mohaghegh P, Salehi S, Mansoori Y (2023). Potential roles of inflammasomes in the pathophysiology of psoriasis: a comprehensive review. Mol Immunol.

[16] Liu T, Wang Q, Du Z, Yin L, Li J, Meng X (2023). The trigger for pancreatic disease: NLRP3 inflammasome. Cell Death Discov.

[17] Song H, Zhao C, Yu Z, Li Q, Yan R, Qin Y (2020). UAF1 deubiquitinase complexes facilitate NLRP3 inflammasome activation by promoting NLRP3 expression. Nat Commun.

[18] Xu H, Chen J, Chen P, Li W, Shao J, Hong S (2023). Costunolide covalently targets NACHT domain of NLRP3 to inhibit inflammasome activation and alleviate NLRP3-driven inflammatory diseases. Acta Pharm Sin B.

[19] Biasizzo M, Kopitar-Jerala N (2020). Interplay between NLRP3 inflammasome and autophagy. Front Immunol.

[20] Hochheiser IV, Behrmann H, Hagelueken G, Rodríguez-Alcázar JF, Kopp A, Latz E (2022). Directionality of PYD filament growth determined by the transition of NLRP3 nucleation seeds to ASC elongation. Sci Adv.

[21] Nong W, Bao C, Chen Y, Wei Z (2022). miR-212-3p attenuates neuroinflammation of rats with Alzheimer’s disease via regulating the SP1/BACE1/NLRP3/Caspase-1 signaling pathway. Bosn J Basic Med Sci.

[22] Jin X, Ma Y, Liu D, Huang Y (2023). Role of pyroptosis in the pathogenesis and treatment of diseases. MedComm (2020).

[23] Faria SS, Costantini S, de Lima VCC, de Andrade VP, Rialland M, Cedric R (2021). NLRP3 inflammasome-mediated cytokine production and pyroptosis cell death in breast cancer. J Biomed Sci.

[24] Gao L, Dong X, Gong W, Huang W, Xue J, Zhu Q (2021). Acinar cell NLRP3 inflammasome and gasdermin D (GSDMD) activation mediates pyroptosis and systemic inflammation in acute pancreatitis. Br J Pharmacol.

[25] de Deus IJ, Martins-Silva AF, Fagundes MMA, Paula-Gomes S, Silva FGDE, da Cruz LL (2023). Role of NLRP3 inflammasome and oxidative stress in hepatic insulin resistance and the ameliorative effect of phytochemical intervention. Front Pharmacol.

[26] Xu J, Núñez G (2023). The NLRP3 inflammasome: activation and regulation. Trends Biochem Sci.

[27] Li S, Wang L, Xu Z, Huang Y, Xue R, Yue T (2021). ASC deglutathionylation is a checkpoint for NLRP3 inflammasome activation. J Exp Med.

[28] Kong X, Zhao Y, Wang X, Yu Y, Meng Y, Yan G (2023). Loganin reduces diabetic kidney injury by inhibiting the activation of NLRP3 inflammasome-mediated pyroptosis. Chem Biol Interact.

[29] Qin Y, Zhao W (2023). Posttranslational modifications of NLRP3 and their regulatory roles in inflammasome activation. Eur J Immunol.

[30] Chen M, He Y, Hu X, Dong X, Yan Z, Zhao Q (2023). Vitamin D3 attenuates SARS-CoV-2 nucleocapsid protein-caused hyperinflammation by inactivating the NLRP3 inflammasome through the VDR-BRCC3 signaling pathway in vitro and in vivo. MedComm (2020).

[31] Bragoszewski P, Turek M, Chacinska A (2017). Control of mitochondrial biogenesis and function by the ubiquitin-proteasome system. Open Biol.

[32] Watanabe Y, Taguchi K, Tanaka M (2020). Ubiquitin, autophagy and neurodegenerative diseases. Cells.

[33] van Wijk SJ, Fulda S, Dikic I, Heilemann M (2019). Visualizing ubiquitination in mammalian cells. EMBO Rep.

[34] Komander D, Rape M (2012). The ubiquitin code. Annu Rev Biochem.

[35] Akther M, Haque ME, Park J, Kang TB, Lee KH (2021). NLRP3 Ubiquitination—A new approach to target NLRP3 Inflammasome Activation. Int J Mol Sci.

[36] Alcaide-Loridan C, Jupin I (2012). Ubiquitin and plant viruses, let’s play together!. Plant Physiol.

[37] Komander D, Clague MJ, Urbé S (2009). Breaking the chains: structure and function of the deubiquitinases. Nat Rev Mol Cell Biol.

[38] Hang Y, Tan L, Chen Q, Liu Q, Jin Y (2021). E3 ubiquitin ligase TRIM24 deficiency promotes NLRP3/caspase-1/IL-1β-mediated pyroptosis in endometriosis. Cell Biol Int.

[39] Yu Q, Shi H, Ding Z, Wang Z, Yao H, Lin R (2023). The E3 ubiquitin ligase TRIM31 attenuates NLRP3 inflammasome activation in helicobacter pylori-associated gastritis by regulating ROS and autophagy. Cell Commun Signal.

[40] Shen J, Wu Q, Liang T, Zhang J, Bai J, Yuan M (2021). TRIM40 inhibits IgA1-induced proliferation of glomerular mesangial cells by inactivating NLRP3 inflammasome through ubiquitination. Mol Immunol.

[41] Li J, Lin H, Fan T, Huang L, Zhang X, Tai Y (2023). BPOZ-2 is a negative regulator of the NLPR3 inflammasome contributing to SARS-CoV-2-induced hyperinflammation. Front Cell Infect Microbiol.

[42] Yan H, Zhou T, Wang Y, Liu Z, Ali I, Sheng L (2023). CDK5RAP3, a key defender of udder, modulates NLRP3 inflammasome activation by regulating autophagolysosome degradation in S. Agalactiae-infected mastitis. Int J Biol Macromol.

[43] Guo Y, Li L, Xu T, Guo X, Wang C, Li Y (2020). HUWE1 mediates inflammasome activation and promotes host defense against bacterial infection. J Clin Invest.

[44] Liu X, Lei Q (2020). TRIM62 knockout protects against cerebral ischemic injury in mice by suppressing NLRP3-regulated neuroinflammation. Biochem Biophys Res Commun.

[45] Ni J, Guan C, Liu H, Huang X, Yue J, Xiang H (2021). Ubc13 promotes K63-linked polyubiquitination of NLRP3 to activate inflammasome. J Immunol.

[46] Wang Q, Qiu H (2023). Deubiquitinase USP16 induces gouty arthritis via Drp1-dependent mitochondrial fission and NLRP3 inflammasome activation. Arthritis Res Ther.

[47] Liu X, Fang Y, Lv X, Hu C, Chen G, Zhang L (2023). Deubiquitinase OTUD6A in macrophages promotes intestinal inflammation and colitis via deubiquitination of NLRP3. Cell Death Differ.

[48] Xiang Y, Li X, Cai M, Cai D (2023). USP9X promotes lipopolysaccharide-stimulated acute lung injury by deubiquitination of NLRP3. Cell Biol Int.

[49] Hai B, Mao T, Du C, Jia F, Liu Y, Song Q (2022). USP14 promotes pyroptosis of human annulus fibrosus cells derived from patients with intervertebral disc degeneration through deubiquitination of NLRP3. Acta Biochim Biophys Sin (Shanghai).

[50] Ramachandran A, Kumar B, Waris G, Everly D (2021). Deubiquitination and activation of the NLRP3 inflammasome by UCHL5 in HCV-infected cells. Microbiol Spectr.

[51] Gong L, Han Y, Chen R, Yang P, Zhang C (2022). LncRNA ZNF883-mediated NLRP3 inflammasome activation and epilepsy development involve USP47 upregulation. Mol Neurobiol.

[52] Bednash JS, Johns F, Patel N, Smail TR, Londino JD, Mallampalli RK (2021). The deubiquitinase STAMBP modulates cytokine secretion through the NLRP3 inflammasome. Cell Signal.

[53] Zhang W, Tao SS, Wang T, Zhang J, Liu X, Li YT (2021). ABRO1 stabilizes the deubiquitinase BRCC3 through inhibiting its degradation mediated by the E3 ubiquitin ligase WWP2. FEBS Lett.

[54] Li X, Wang T, Tao Y, Wang X, Li L, Liu J (2022). MF-094, a potent and selective USP30 inhibitor, accelerates diabetic wound healing by inhibiting the NLRP3 inflammasome. Exp Cell Res.

[55] Qiu T, Guo J, Wang L, Shi L, Ai M, Xia Z (2022). Dynamic microglial activation is associated with LPS-induced depressive-like behavior in mice: an [18F] DPA-714 PET imaging study. Bosn J Basic Med Sci.

[56] Wang Z, Zhang S, Xiao Y, Zhang W, Wu S, Qin T (2020). NLRP3 inflammasome and inflammatory diseases. Oxid Med Cell Longev.

[57] Sakshi S, Jayasuriya R, Ganesan K, Xu B, Ramkumar KM (2021). Role of circRNA-miRNA-mRNA interaction network in diabetes and its associated complications. Mol Ther Nucleic Acids.

[58] Halim M, Halim A (2019). The effects of inflammation, aging and oxidative stress on the pathogenesis of diabetes mellitus (type 2 diabetes). Diabetes Metab Syndr.

[59] Wang B, Dai Z, Gao Q, Liu Y, Gu G, Zheng H (2022). Spop ameliorates diabetic nephropathy through restraining NLRP3 inflammasome. Biochem Biophys Res Commun.

[60] Wei C, Xu J, Liu Y, Qadir J, Zhang S, Yuan H (2023). Exogenous spermidine alleviates diabetic myocardial fibrosis via suppressing inflammation and pyroptosis in db/db Mice. Balkan Med J.

[61] Zhang J, Chen C, Wu L, Wang Q, Chen J, Zhang S (2022). Synoviolin inhibits the inflammatory cytokine secretion of Müller cells by reducing NLRP3. J Mol Endocrinol.

[62] Ruan Z, Li Y, Chen Y (2022). HECTD3 promotes NLRP3 inflammasome and pyroptosis to exacerbate diabetes-related cognitive impairment by stabilising MALT1 to regulate JNK pathway. Arch Physiol Biochem.

[63] Dugger BN, Dickson DW (2017). Pathology of neurodegenerative diseases. Cold Spring Harb Perspect Biol.

[64] Chen WW, Zhang X, Huang WJ (2016). Role of neuroinflammation in neurodegenerative diseases (Review). Mol Med Rep.

[65] Zhu Z, Huang P, Sun R, Li X, Li W, Gong W (2022). A novel long-noncoding RNA LncZFAS1 prevents MPP+-induced neuroinflammation through MIB1 activation. Mol Neurobiol.

[66] Yan YQ, Zheng R, Liu Y, Ruan Y, Lin ZH, Xue NJ (2023). Parkin regulates microglial NLRP3 and represses neurodegeneration in Parkinson’s disease. Aging Cell.

[67] Han X, Sun S, Sun Y, Song Q, Zhu J, Song N (2019). Small molecule-driven NLRP3 inflammation inhibition via interplay between ubiquitination and autophagy: implications for Parkinson disease. Autophagy.

[68] Suresh P, Jasmin S, Yen Y, Hsu HJ, Varinthra P, Pairojana T (2022). Attenuation of HECT-E3 ligase expression rescued memory deficits in 3xTg-AD mice. Front Aging Neurosci.

[69] Whittaker JL, Truong LK, Dhiman K, Beck C (2021). Osteoarthritis year in review 2020: rehabilitation and outcomes. Osteoarthritis Cartilage.

[70] Xie C, Chen Q (2019). Adipokines: New therapeutic target for osteoarthritis?. Curr Rheumatol Rep.

[71] Chen Y, Liu Y, Jiang K, Wen Z, Cao X, Wu S (2022). Linear ubiquitination of LKB1 activates AMPK pathway to inhibit NLRP3 inflammasome response and reduce chondrocyte pyroptosis in osteoarthritis. J Orthop Translat.

[72] Sun D, Peng Y, Ge S, Fu Q (2022). USP1 inhibits NF-κB/NLRP3 induced pyroptosis through TRAF6 in osteoblastic MC3T3-E1 cells. J Musculoskelet Neuronal Interact.

[73] Liu G, Liu Q, Yan B, Zhu Z, Xu Y (2021). USP7 inhibition alleviates H2O2-induced injury in chondrocytes via inhibiting NOX4/NLRP3 pathway. Front Pharmacol.

[74] Qian J, Wang X, Su G, Shu X, Huang Z, Jiang H (2022). Platelet-rich plasma-derived exosomes attenuate intervertebral disc degeneration by promoting NLRP3 autophagic degradation in macrophages. Int Immunopharmacol.

[75] Kaplan GG, Windsor JW (2021). The four epidemiological stages in the global evolution of inflammatory bowel disease. Nat Rev Gastroenterol Hepatol.

[76] Zhou L, Liu T, Huang B, Luo M, Chen Z, Zhao Z (2021). Excessive deubiquitination of NLRP3-R779C variant contributes to very-early-onset inflammatory bowel disease development. J Allergy Clin Immunol.

[77] Dai G, Jiang Z, Sun B, Liu C, Meng Q, Ding K (2020). Caffeic acid phenethyl ester prevents colitis-associated cancer by inhibiting NLRP3 inflammasome. Front Oncol.

[78] Yang N, Xia Z, Shao N, Li B, Xue L, Peng Y (2017). Carnosic acid prevents dextran sulfate sodium-induced acute colitis associated with the regulation of the Keap1/Nrf2 pathway. Sci Rep.

[79] Li XY, Yang YT, Zhao Y, Kong XH, Yang G, Hong J (2021). Moxibustion inhibits the expression of colonic NLRP3 through miR7/RNF183/NF-κB signaling pathway in UC Rats. Evid Based Complement Alternat Med.

[80] Wang L, Zhao X, Ding J, Liu Y, Liu H, Zheng L (2023). Oridonin attenuates the progression of atherosclerosis by inhibiting NLRP3 and activating Nrf2 in apolipoprotein E-deficient mice. Inflammopharmacology.

[81] Cheng X, Xu S, Zhang C, Qin K, Yan J, Shao X (2020). The BRCC3 regulated by Cdk5 promotes the activation of neuronal NLRP3 inflammasome in Parkinson’s disease models. Biochem Biophys Res Commun.

[82] Zhang S, Guan X, Liu W, Zhu Z, Jin H, Zhu Y (2022). YTHDF1 alleviates sepsis by upregulating WWP1 to induce NLRP3 ubiquitination and inhibit caspase-1-dependent pyroptosis. Cell Death Discov.

[83] Liu X, Lin Z, Yin X (2022). Pellino2 accelerate inflammation and pyroptosis via the ubiquitination and activation of NLRP3 inflammation in model of pediatric pneumonia. Int Immunopharmacol.

[84] Glick D, Barth S, Macleod KF (2010). Autophagy: cellular and molecular mechanisms. J Pathol.

[85] Cai B, Zhao J, Zhang Y, Liu Y, Ma C, Yi F (2022). USP5 attenuates NLRP3 inflammasome activation by promoting autophagic degradation of NLRP3. Autophagy.

[86] Shao L, Dong C, Geng D, He Q, Shi Y (2022). Ginkgolide B inactivates the NLRP3 inflammasome by promoting autophagic degradation to improve learning and memory impairment in Alzheimer’s disease. Metab Brain Dis.

[87] Park E, Choi H, Truong CS, Jun HS (2023). The inhibition of autophagy and pyroptosis by an ethanol extract of nelumbo nucifera leaf contributes to the amelioration of dexamethasone-induced muscle atrophy. Nutrients.

[88] Tang Y, Zhou X, Cao T, Chen E, Li Y, Lei W (2022). Endoplasmic reticulum stress and oxidative stress in inflammatory diseases. DNA Cell Biol.

[89] Wiegman CH, Li F, Ryffel B, Togbe D, Chung KF (2020). Oxidative stress in ozone-induced chronic lung inflammation and emphysema: a facet of chronic obstructive pulmonary disease. Front Immunol.

[90] Zhu Y, Zhang M, Wang J, Wang Q (2022). Knockdown of UAF1 alleviates sevoflurane-induced cognitive impairment and neurotoxicity in rats by inhibiting pro-inflammatory signaling and oxidative stress. J Toxicol Sci.

[91] Cheng Y, Cheng L, Gao X, Chen S, Wu P, Wang C (2021). Covalent modification of Keap1 at Cys77 and Cys434 by pubescenoside a suppresses oxidative stress-induced NLRP3 inflammasome activation in myocardial ischemia-reperfusion injury. Theranostics.

[92] Cui Y, Yang Y, Tao W, Peng W, Luo D, Zhao N (2023). Neutrophil extracellular traps induce alveolar macrophage pyroptosis by regulating NLRP3 deubiquitination, aggravating the development of septic lung injury. J Inflamm Res.

[93] Li XC, Luo SJ, Fan W, Zhou TL, Tan DQ, Tan RX (2022). Macrophage polarization regulates intervertebral disc degeneration by modulating cell proliferation, inflammation mediator secretion, and extracellular matrix metabolism. Front Immunol.

[94] Yang X, Wang C, Zhu G, Guo Z, Fan L (2023). METTL14/YTHDF1 axis-modified UCHL5 aggravates atherosclerosis by activating the NLRP3 inflammasome. Exp Cell Res.

[95] Shang L, Du Y, Zhao Y, Zhang Y, Liu C (2023). The interaction of OTUB1 and TRAF3 mediates NLRP3 inflammasome activity to regulate TGF-β1-induced BEAS-2B cell injury. Appl Biochem Biotechnol.

